# Silybin restores glucose uptake after tumour necrosis factor-alpha and lipopolysaccharide stimulation in 3T3-L1 adipocytes

**DOI:** 10.1080/21623945.2024.2374062

**Published:** 2024-07-02

**Authors:** Alejandra Butanda-Nuñez, Octavio Rodríguez-Cortés, Espiridión Ramos-Martínez, Marco Antonio Cerbón, Galileo Escobedo, Anahí Chavarría

**Affiliations:** a Unidad de Medicina Experimental, Facultad de Medicina, Universidad Nacional Autónoma de México, Mexico City, Mexico; b Laboratorio 103, SEPI, Escuela Superior de Medicina, Instituto Politécnico Nacional, Mexico City, Mexico; c Unidad de Investigación en Reproducción Humana, Instituto Nacional de Perinatología-Facultad de Química, Universidad Nacional Autónoma de México, Mexico City, Mexico; d Laboratorio de Proteómica y Metabolómica, Hospital General de México “Dr. Eduardo Liceaga”, Mexico City, Mexico

**Keywords:** 3T3-L1 adipocytes, glucose uptake, inflammatory stimuli, LPS, silybin, TNFα

## Abstract

Obesity is associated with a low-grade chronic inflammatory process characterized by higher circulating TNFα levels, thus contributing to insulin resistance. This study evaluated the effect of silybin, the main bioactive component of silymarin, which has anti-inflammatory properties, on TNFα levels and its impact on glucose uptake in the adipocyte cell line 3T3-L1 challenged with two different inflammatory stimuli, TNFα or lipopolysaccharide (LPS). Silybin’s pre-treatment effect was evaluated in adipocytes pre-incubated with silybin (30 or 80 µM) before challenging with the inflammatory stimuli (TNFα or LPS). For the post-treatment effect, the adipocytes were first challenged with the inflammatory stimuli and then post-treated with silybin. After treatments, TNFα production, glucose uptake, and GLUT4 protein expression were determined. Both inflammatory stimuli increased TNFα secretion, diminished GLUT4 expression, and significantly decreased glucose uptake. Silybin 30 µM only reduced TNFα secretion after the LPS challenge. Silybin 80 µM as post-treatment or pre-treatment decreased TNFα levels, improving glucose uptake. However, glucose uptake enhancement induced by silybin did not depend on GLUT4 protein expression. These results show that silybin importantly reduced TNFα levels and upregulates glucose uptake, independently of GLUT4 protein expression.

## 1. Introduction

Obesity is a systemic, chronic, and multifactorial disease, with a global epidemic rise in almost all countries [1], which is related to metabolic alterations like insulin resistance, contributing to the development of chronic diseases like diabetes mellitus type 2 [2,3]. Insulin resistance is an insufficient cellular response to insulin in insulin-dependent cells like adipocytes, cardiomyocytes, and skeletal muscle [4,5]. Several studies have shown specific molecular mechanisms involved in the pathophysiology of insulin resistance [6]. Low-grade chronic inflammation associated with obesity correlates with insulin resistance [7,8], particularly tumour necrosis factor α (TNFα), secreted by adipocytes and adipose tissue macrophages, impairs the insulin signalling via serine phosphorylation of insulin receptor substrate 1 (IRS1), reducing glucose transporter 4 (GLUT4) membrane expression, therefore decreasing glucose entry into cells [9,10]. Another mechanism involved is metabolic endotoxemia, characterized by increased circulating lipopolysaccharide (LPS) or the LPS binding protein, a phenomenon known as metabolic endotoxemia [11].

Currently, therapies are based on preventing the development of diseases associated with obesity. Silymarin is a compound of flavonolignans obtained from *Silybum marianum*, a European native plant, which has been traditionally used as a hepatoprotector [12]. Silybin is the main active component of silymarin and represents a therapeutic option due to its antioxidant and anti-inflammatory properties, proven in different experimental models and clinical studies [13–17]. Although the molecular bases of the anti-inflammatory and immunomodulatory effects of silymarin are still not fully understood, several reports demonstrated that silybin inhibits the phosphorylation of the transcription factor nuclear factor kappa-light-chain-enhancer of activated B cells (NFκB), which regulates and coordinates transcription and expression of several genes involved in the inflammatory processes [18–20]. Also, silybin enhances the sensitivity to insulin of insulin-dependent cells like hepatocytes and myocytes [21,22] and has a hypoglycaemic effect in patients with diabetes mellitus type 2 [16].

**Figure 1.**
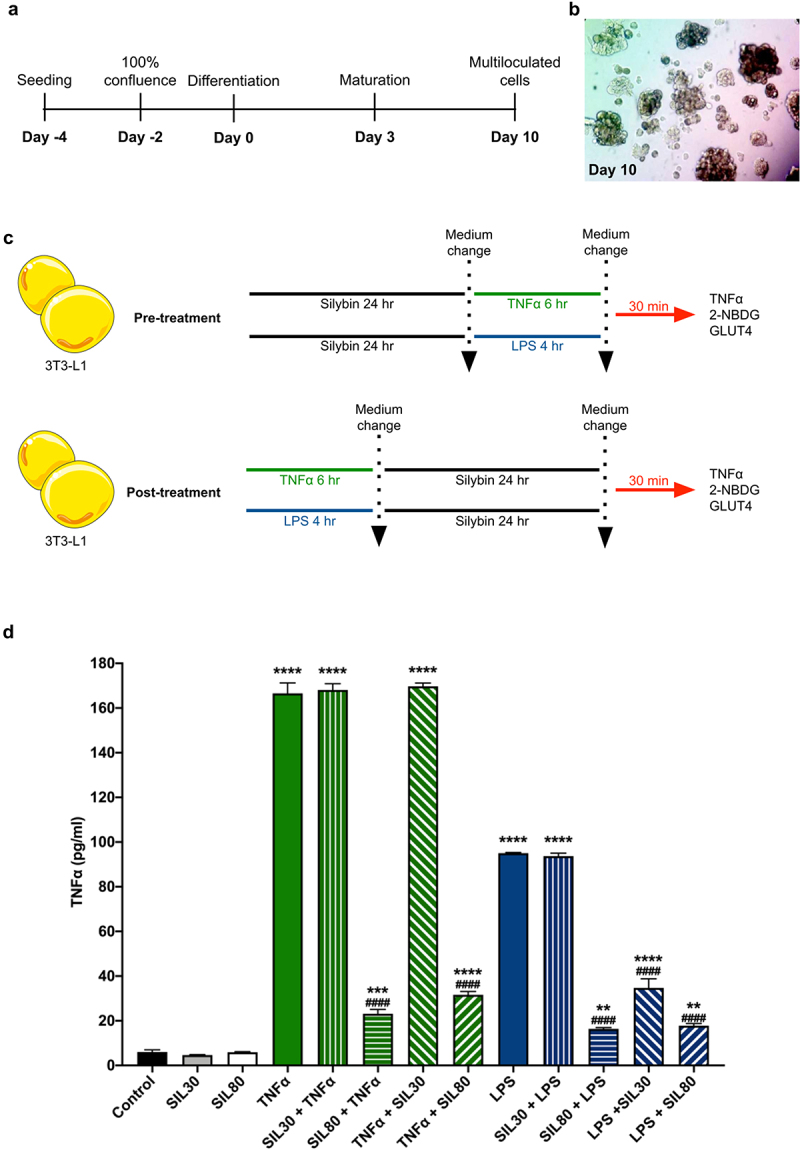
Experimental design and TNFα secretion with silybin treatment. (a) 3T3-L1 preadipocyte differentiation protocol. (b) Representative image of 3T3-L1 preadipocytes at day 10 of induction. (c) For silybin’s pre-treatment, 3T3-L1 adipocytes were pre-treated with silybin and then challenged with TNFα or LPS. For silybin’s post-treatment, 3T3-L1 adipocytes were first challenged with TNFα or LPS and then post-treated with silybin. (d) TNFα supernatant levels after treating 3T3-L1 adipocytes with vehicles (Control), silybin 30 µM (SIL30), silybin 80 µM (SIL80), TNFα (TNFα), LPS (LPS), silybin then stimulated with TNFα (SIL30 + TNFα, SIL80 + TNFα) or LPS (SIL30 + LPS, SIL80 + LPS), challenged with TNFα then post-treated with silybin (TNFα + SIL30, TNFα + SIL80), or LPS and then post-treated with silybin (LPS + SIL30, LPS + SIL80). Data represented mean ± SEM from five independent experiments and were analysed by one-way ANOVA, followed by a Dunnett’s post hoc test. *p ≤ 0.01 compared to the control group. #*p* < 0.0001 compared to the TNFα or LPS groups.

Since obesity is a chronic inflammatory state mainly generated by the adipose tissue and is a primary cause for the development of metabolic diseases such as diabetes mellitus, it is relevant to explore if silybin exerts a hypoglycaemic and anti-inflammatory effect in insulin-sensitive cells such as adipocytes. In this study, we, therefore, evaluated the impact of silybin as pre-treatment (preventive) and post-treatment (therapeutic) on TNFα levels and its impact on glucose uptake in the adipocyte cell line 3T3-L1 challenged with two different inflammatory stimuli, TNFα and LPS (Figure 1).

## 2. Results

### 2.1. Silybin treatment decreases TNFα expression after inflammatory stimuli

One of the most explored effects of silybin is its anti-inflammatory effect. Silybin inhibits TNFα synthesis even when administered as pre-treatment by inhibiting the inhibitor of the nuclear factor kappa B (IκBα) phosphorylation, proteolytic degradation, and thus NFκB translocation to the nucleus [18,23–25].

Both inflammatory stimuli, TNFα and LPS, significantly increased TNFα levels in the supernatants of the 3T3-L1 adipocyte cells (*p* < 0.0001; Figure 1d). Interestingly, TNFα stimulation induced significantly higher levels of TNFα than LPS stimulation (*p* < 0.0001; Figure 1d). Silybin in 30 and 80 µM doses did not affect basal TNFα levels (Figure 1d). Silybin at the dose of 30 µM did not modify TNFα levels, both as pre-treatment and post-treatment, on TNFα stimulation (Figure 1d). However, the pre-treatment and post-treatment with silybin 80 µM reduced TNFα secretion significantly compared to the TNFα challenged group (*p* < 0.0001; Figure 1d). Silybin as 30 µM pre-treatment did not change TNFα secretion when the inflammatory stimulus was LPS, but TNFα lowered when silybin was administered as post-treatment (*p* < 0.0001; Figure 1d). Silybin 80 µM treatment before and after the LPS stimuli reduced TNFα levels in the supernatant significantly in a similar manner to the control group (*p* < 0.0001; Figure 1d).

### 2.2. Silybin increases the uptake of glucose

**Figure 2.**
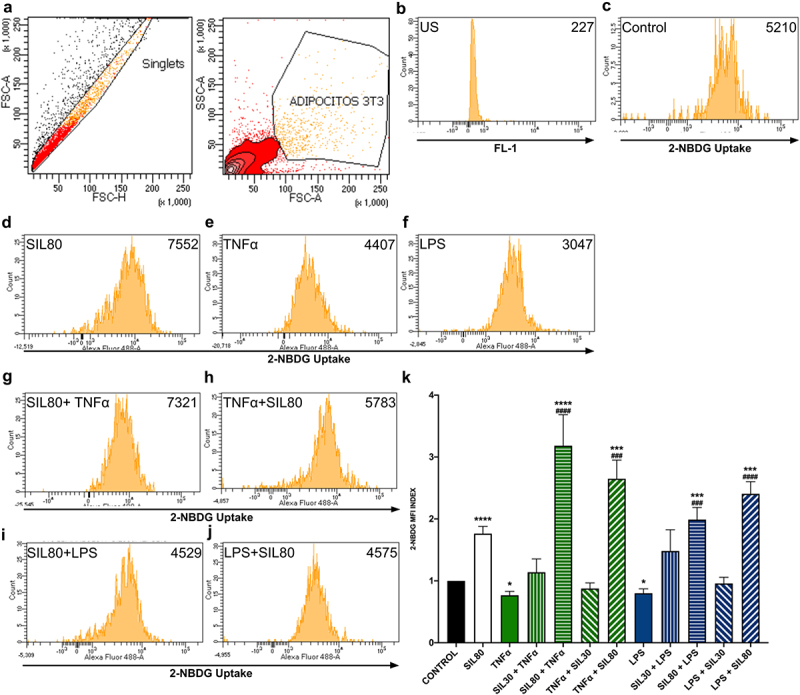
Effect of silybin on glucose uptake by 3T3-L1 adipocytes. (a) Flow cytometric analysis for 3T3-L1 adipocytes. (b) Auto-fluorescence of unstained cells (US) on FL-1 channel. (c) The control was 3T3-adipocytes cultured using only a maturation medium and vehicles. Representative flow cytometry histograms showing 2-NBDG uptake in 3T3-L1 adipocytes after treatments: (d) silybin (SIL80), (e) TNFα, (f) LPS, (g) pre-treatment with silybin and then stimulated with TNFα (SIL80 + TNFα), (i) pre-treatment with silybin and then LPS (SIL80 + LPS), (h) challenged with TNFα and then post-treated with silybin (TNFα + SIL80), (j) challenged with LPS and then post-treated with silybin (LPS + SIL80). Since 30 µM of silybin had no effect on glucose uptake, only representative flow cytometry histograms for 80 µM of silybin are presented. (k) 2-NBDG uptake was by 3T3-L1 adipocytes after treatments with inflammatory stimuli, pre- and post-treated with silybin 30 µM (SIL30) or 80 µM (SIL80). The control group received only a maturation medium and vehicles (control value set at 1). Data represented mean ± SEM from 3 independent experiments carried out in triplicate and were analysed by one-way ANOVA, followed by a Dunnett’s post hoc test. *p ≤ 0.05 compared to the control group, #*p* ≤ 0.01 compared to TNFα or LPS groups. The MFI index was calculated by dividing the MFI-treated group by the MFI-negative control.

Silybin at the dose of 30 µM did not increase glucose uptake, either as pre-treatment or post-treatment with TNF or LPS challenges (Figure 2k). Silybin (80 µM) treatment raised glucose uptake significantly (*p* < 0.0001; Figures 2d,k). The stimulation of the adipocytes with TNFα or LPS decreased glucose analog 2NBDG uptake when compared to the control (*p* = 0.01; *p* = 0.028, respectively; Figures 2e,f,k). The pre-treatment with silybin 80 µM raised glucose uptake when compared to the control group (SIL80 + TNFα *p* < 0. 0001, SIL80 + LPS *p* = 0.0007; Figures 2g,i,k) and their respective TNFα and LPS control groups (*p* < 0.0001, *p* = 0.0002, respectively; Figures 2g,i,k). Also, silybin 80 µM as post-treatment after both inflammatory stimuli increased glucose uptake when compared to the control group (*p* = 0.0002, *p* < 0. 0001, respectively; Figures 2g,i,k) and to their respective TNFα and LPS control groups (*p* = 0.0002, *p* < 0.0001, respectively; Figures 2g,i,k). Silybin’s effect on glucose uptake is probably due to TNFα reduction, thus improving insulin receptor signalling and glucose uptake.

### 2.3. Inflammatory stimuli reduce GLUT4 protein expression

Since 30 µM of silybin, neither pre-treatment nor post-treatment showed no modulatory effect on secreted TNFα levels nor 2NBDG uptake after inflammatory stimuli challenges (Figures 1b and 2k), the GLUT4 experiments were only performed with the dose of 80 µM.

**Figure 3.**
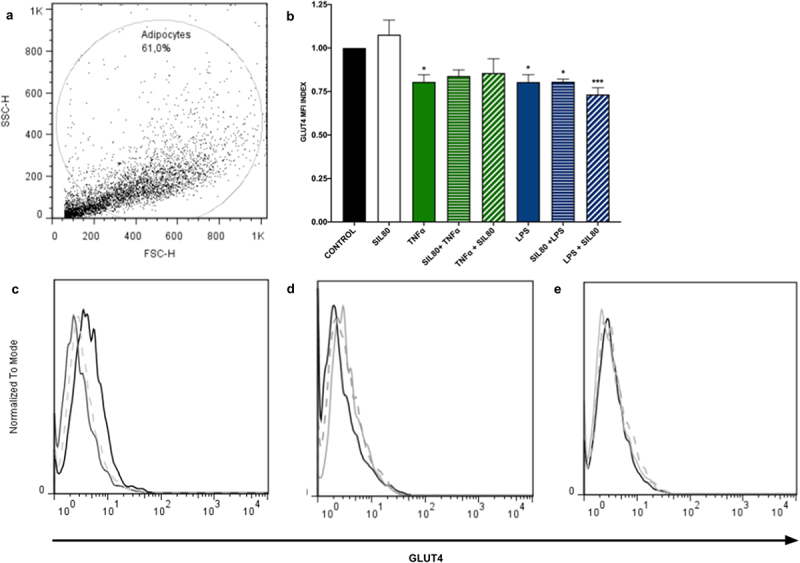
GLUT4 protein expression is not modified with silybin treatment. (a) Flow cytometric analysis of 3T3-L1 adipocytes. (b) GLUT4 protein expression by 3T3-adipocytes after treatment with 80 μM of silybin (SIL80), TNFα, LPS, pre-treated with silybin and then stimulated with TNFα (SIL80 + TNFα) or LPS (SIL80 + LPS), challenged with TNFα and then post-treated with silybin (TNFα + SIL80), or challenged with LPS and then post-treated with silybin (LPS + SIL80). The control group received only a maturation medium and vehicles (control value set at 1). Data represented mean ± SEM from 5 independent experiments and were analysed by one-way ANOVA, followed by a Dunnett’s post hoc test. *p ≤ 0.005 compared to the control group. The MFI index was calculated by dividing the MFI-treated group by the MFI-negative control. (c) Representative flow cytometry histograms showing GLUT4 protein expression in 3T3-L1 adipocytes after treatment with 80 μM of silybin (SIL; solid black line), TNFα (solid grey line), or LPS (dashed grey line). (d) Representative flow cytometry histograms showing GLUT4 protein expression in 3T3-L1 adipocytes after treatment with TNFα (solid black line), pre-treated with silybin and then challenged with TNFα (SIL80 + TNFα; dashed grey line), or treated with TNFα and then post-treated with silybin (TNFα + SIL80; solid grey line). (e) Representative flow cytometry histograms showing GLUT4 protein expression in 3T3-L1 adipocytes after treatment with LPS (solid black line), pre-treated with silybin and then challenged with LPS (SIL80 + LPS; dashed grey line), or treated with LPS and then post-treated with silybin (LPS + SIL80; solid grey line).

Inflammation plays a crucial role in down-regulating the expression of glucose transport GLUT4 [26]. Our data showed that exposure to inflammation significantly reduces GLUT4 protein expression in adipocytes (TNFα *p* = 0.0177, LPS *p* = 0.0171; Figure 3b,c). Silybin pre- nor post-treatment had no effect on GLUT4 protein expression (Figure 3).

## 3. Discussion

Inflammation can be triggered by external disruptors such as pathogens (e.g. bacteria), intrinsic events like cell death, cancer, or physiological alterations secondary to fasting or overfeeding [27]. Adipose tissue plays an important link between inflammation and metabolism. Indeed, during obesity, the endotoxins cross the intestinal barrier, inducing multiple immune alterations in humans [28–30]. In mice, a high-fat diet reduces bifidobacteria and increases the plasma concentration of LPS and liver fat, along with the expression of several pro-inflammatory cytokines like IL6 and TNFα [31]. Abundant evidence recently relates obesity and over-nutrition to a chronic low-grade inflammatory state caused by immune cells and adipocytes; this process is called ‘meta-inflammation’ [32,33].

The role of the adipocyte is more than the storage of lipids; extensive evidence demonstrates its participation in the regulation and alterations of glucose metabolism. Adipose tissue acts as a producer and consumer of multiple inflammatory mediators such as TNFα, IL-1β, and IL-6 [7,34]. TNFα was the first pro-inflammatory cytokine related to inflammation-induced insulin resistance [10]. As demonstrated by other reports, we observed that the incubation of adipocytes with TNFα or LPS induced the production of TNFα (Figure 1d) [35,36]. Interestingly, pre- and post-incubation with silybin 80 µM decreased TNFα levels significantly (Figure 1d). Silybin’s and silymarin’s inhibitory effect of the transcription factor nuclear factor kappa B (NFkB) and c-Jun N-terminal kinase (JNK) has been widely documented in the brain [37–39], lung [40,41], liver [42–47], pancreas [19], and even adipocytes [48,49]. Probably, silybin decreased the phosphorylation of NFkB in the 3T3-L1 adipocytes, thereby decreasing the transcription of inflammatory genes and thus reducing TNFα in the supernatant after both inflammatory stimuli.

Insulin resistance is a pathologic condition in which adipocytes, hepatocytes, and skeletal muscle cells, insulin‐dependent cells, fail to respond appropriately to physiological circulating insulin levels [4]. Since insulin plays a crucial role in cell glucose entry, any disruption in insulin signal transduction interferes with glucose uptake by the cells, thus leading to hyperglycaemia [4]. Insulin resistance is a common feature of obesity, non-alcoholic fatty liver disorder, diabetes mellitus type 2, dyslipidemia, atherosclerosis, and hypertension, all metabolic disorders [4,50]. Our data showed that the TNFα and LPS stimuli reduced glucose uptake, most likely because both challenges decreased the expression of GLUT4 in 3T3-L1 adipocytes (Figure 3) [51]. TNFα is crucial in insulin resistance, as it interferes with insulin receptor signalling by phosphorylating ISR1/2, inhibiting Akt, and therefore diminishing the translocation of GLUT4 to the plasma membrane [52,53]. Despite not having determined Akt phosphorylation, the doses of both stimuli used in this study have been shown to inhibit Akt phosphorylation [51,54]; it is very likely that in our model, both inflammatory stimuli also interfere with membranal translocation of GLUT4 via inhibition of Akt phosphorylation. Also, activation of JNK seems to be involved in TNFα-induced insulin resistance [55]. NFkB, a transcription factor activated by TNFα or LPS, and JNK signalling pathways are targets of silybin. Our results demonstrate that silybin 80 µM as pre- and post-treatment increases glucose uptake by counteracting the effect induced by these inflammatory stimuli.

Silybin 80 µM alone, neither pre-treatment nor post-treatment, did not modify GLUT4 expression in adipocytes as previously reported (Figure 3) [56,57]. The discrepancies are probably due to the different doses and times of incubation of silybin employed. We exposed the adipocytes to 24 hr of silybin in 30 and 80 µM as pre-treatment and post-treatment, while Nomura and colleagues used 10 µM 1 hr, and Zhan and colleagues 40 µM 20 min before glucose uptake assay [56,57]. Another key difference may be that both works explored the effect of silybin alone; here, we observed that silybin enhanced glucose uptake even after TNFα and LPS lowered glucose capture by the adipocytes (Figure 2).

Being GLUT4, one of the primary glucose transporters, we expected that by modulating the inflammation with silybin, GLUT4 would increase its expression. However, silybin, as pre-treatment and post-treatment, showed no significant changes in the expression of GLUT4 (Figures 3b,d,e). Although GLUT4 transporter levels did not increase with silybin treatment (Figure 3), we cannot discard that membrane translocation is happening like in other flavonoids, epigallocatechin Gallate or kaempferol, among others, which increase GLUT4 membrane translocation in adipocytes after treatments [58–60].

Another possibility is that the higher glucose uptake is due to the activity of another transporter, like Glucose transporter type 12 (GLUT12) [61]. GLUT12 is a second insulin-responsive glucose transporter in the white adipose and skeletal tissue [61,62]. However, unlike GLUT4 and Glucose transporter type 1(GLUT1), GLUT12 does not decrease due to inflammatory stimuli such as hypoxia or TNFα [61,63,64]. It seems that GLUT12 could compensate for the inflammation-induced down-regulation of GLUT4 in adipose tissue of obese mice and 3T3-L1 adipocytes [61]. Possibly, silybin in our model could induce GLUT12 expression by unknown mechanisms.

We also cannot rule out that silybin’s beneficial effect on glucose uptake is similar to other plant flavonoids that have an additional effect on PPARγ [65,66] and adiponectin expression levels [67]. Under inflammatory conditions, TNFα reduces PPARγ expression, while its blockade restores PPARγ levels and functions on adipokines like adiponectin in 3T3-L1 adipocyte cells, increasing glucose uptake [65–67].

In conclusion, our data show that inflammatory stimuli like TNFα and LPS increase TNFα levels in the supernatant, lowering glucose uptake significantly due to the decrease in membrane GLUT4 expression, simulating an insulin-resistance *in vitro* model [68,69]. Silybin improves this alteration by diminishing TNFα levels and upregulating glucose uptake by 3T3-L1 cells in the prophylactic and therapeutic administrations. However, this improvement of the insulin resistance state was not modulated by GLUT4 expression. More studies are needed to elucidate further the possible mechanisms involved in improving insulin resistance by silybin.

## 4. Materials and methods

### 4.1. 3T3-L1 cell culture

Twelve thousand five hundred 3T3-L1 cells were cultured in 24-well plates in complete medium [RPMI-1640 (R1383; Sigma-Aldrich) supplemented with 5 mmol of glucose and 10% foetal bovine serum (FBS)], and 1% penicillin–streptomycin mixed solution in a humidified atmosphere at 37°C and 5% CO_2_. Cells reached confluence after two days; two days later, cells received differentiation medium (RPMI-1640, 10% FBS, 1 μM dexamethasone, 1 μM insulin, and 0.5 mM isobutylmethylxanthine) to induce 3T3-L1 differentiation (Day 0). Three days later, the medium was replaced with a maturation medium (complete medium with 1 μM insulin; Day 3), which was changed every second day. After ten days of initiating the differentiation, adipocytes presented multiloculated lipid vacuoles (Day 10; Figure 1a,b).

### 4.2. Inflammatory challenges and silybin treatment

For the preventive effect of silybin, 3T3-L1 cells were pre-treated with 30 or 80 µM of silybin (S0417 Sigma) in DMSO for 24 h in the maturation medium [70,71]. The DMSO final concentration for silybin treatments was less than 0.01% [72]. The cell medium was replaced after cells were washed five times with RPMI supplemented with FBS 10%. The cells were then challenged with 2 ng/mL of lipopolysaccharide (LPS; L4391 Sigma) for 4 h [73,74] or 2 ng/mL of recombinant mouse TNFα (29-8321-65 Invitrogen, ThermoFisher) for 6 h [35,75] in maturation medium (Figure 1c, pre-treatment).

The potential therapeutical effect of silybin was evaluated by first challenging the cells with the inflammatory stimuli (LPS or TNFα) for 4 and 6 h, respectively; cells were washed, cell medium was replaced, and cells were treated with 30 or 80 µM of silybin for 24 h (Figure 1d, post-treatment).

Control cells received either the vehicles DMSO and sterile water, TNFα, LPS, silybin 30 µM, or silybin 80 µM.

After incubation with TNFα, LPS, or silybin, the cells were washed and incubated with fresh maturation medium for another 30 min. The cells were recovered for the glucose uptake or GLUT4 protein expression assays and the cell supernatants for TNFα determination.

### 4.3. TNFα determination by ELISA

The levels of TNFα were measured in cell supernatant by sandwich ELISA (TNFα Mouse ELISA Kit 88–7324; eBioscience). Samples were incubated for 18 h at four °C with PBS-Tween 20 (0.05%)-0.5% BSA, washed three times, and incubated with the corresponding detection antibody for two h at room temperature. The reaction was detected using TMB as the substrate. Optical density readings were done at 450 nm in a spectrophotometer (EPOCH, BioTek). All assays were performed by duplicated, and the assay sensitivity was 3.13 pg/mL.

### 4.4. Glucose uptake

To analyse glucose uptake, the fluorescent glucose analog, 2-(*N*-[7-nitrobenz-2-oxa-1,3-diazol-4-yl] amino)-2-deoxyglucose (2-NBDG; N13195 Invitrogen, Thermo Fisher Scientific), was used for direct quantification of glucose incorporation in living cells by flow cytometry [76,77]. After the challenges, the cells were washed and then transferred to cytometry tubes with phosphate-buffered saline (PBS) containing glucose 0.25 mM and insulin ten μIU/mL in the presence of 100 µM 2-NBDG. After 40 min incubation, cells were washed twice with PBS, trypsinized, centrifuged, and resuspended in PBS, and 150 µL of 4% paraformaldehyde was added. Ten thousand events were analysed in a FACSaria fusion 2 L 8C (Becton Dickinson), and 2-NBDG was measured using the green fluorescence (520 nm) channel FL1. The 3T3L1 adipocytes represented more than 60% of the 10,000 analysed events in all experiments, and 2-NBDG determinations were only made in the adipocyte region. Duplicated events and detritus were excluded. Results were expressed as an index of mean fluorescence intensity (MFI) of treated samples divided by the MFI of control samples (only treated with vehicles).

### 4.5. GLUT4 detection

With some modifications, GLUT4 protein expression was determined with flow cytometry (Koshy et al., 2010). After treatments, cells were washed in PBS without Ca^2+^ and Mg^2+^, with 2% FBS and 0.002% sodium azide added (FACS buffer). Cells were then centrifuged, suspended in the FACS buffer, and incubated for 20 min at 4°C with the anti-GLUT4 antibody (dilution 1:50, AB48547, Abcam). The cells were washed and incubated with the Alexa-488-secondary antibody (dilution 1:40, AB150115, Abcam) on ice for 40 min in the dark. After the incubation, 1 mL washing buffer for FACS was added to each sample. The samples were then vortexed and centrifuged, the supernatant was removed, and a 0.5 mL washing buffer for FACS was added with 1% formaldehyde. Without the primary antibody, the control samples were treated only with the Alexa-488-secondary antibody anti-rabbit IgG (AB150073, Abcam). Each experiment analysed a minimum of 10,000 cells per treatment condition. The 3T3L1 adipocytes represented more than 60% of the 10,000 analysed events in all experiments, and GLUT4 determinations were only made in the adipocyte region. Gates were set to exclude nonviable cells, cell debris, and duplicated events. Results were expressed as an index of MFI of stained samples/MFI control samples.

### 4.6. Statistical analysis

The experimental data are presented as mean values ± SEM from 5 to 10 biological replicates carried out in at least three independent experiments. The results were processed in GraphPad Prism 9. Statistical differences between experimental groups were determined using a one-way analysis of variance test (ANOVA), followed by post hoc Dunnett’s test. *p* ≤ 0.05 was considered significant.

## Data Availability

The data supporting this study’s findings are available from the corresponding authors upon reasonable request.
